# Supplementary material for: Opposing kinesin complexes queue at plus tips to ensure microtubule catastrophe at cell ends
Source: EMBO Rep. 2018 Sep 11;19(11):e46196. doi: 10.15252/embr.201846196 (PMC6216294; doi:10.15252/embr.201846196)
Supplement: Supplementary file 1 — Appendix [file EMBR-19-e46196-s001.pdf]

Opposing kinesin complexes queue at plus tips to ensure microtubule catastrophe at cell ends

Appendix Contents

Page 1 - Appendix Table S1. List of strains used in this study

Page 4 - Appendix Table S2. List of oligonucleotides used in this study

Appendix Table S1. List of strains used in this study

**Figure 1B**		
JM8018	*leu1-32::SV40-GFP-atb2:leu1* h^-	F. Chang
JM8427	*klp5::natR klp6::hygR leu1-32::SV40-GFP-atb2:leu1* h^-	this study
JM8334	*mcp1::kanR leu1-32::SV40-GFP-atb2:leu1* h^+	this study
JM8547	*mcp1::kanR klp5::natR klp6::hygR leu1-32::SV40-GFP-atb2:leu1* $h^?$	this study
**Figure 1C-E**		
JM6823	*natR:Z:adh15-mCherry-atb2* h^-	K. Sawin
JM9441	*klp5-mNeonGreen:kanR klp6-mNeonGreen:hygR natR:Z:adh15-mCherry-atb2* h^+	this study
JM9877	*mcp1::natR klp5-mNeonGreen:kanR klp6-mNeonGreen:hygR natR:Z:adh15-mCherry-atb2* $h^?$	this study
**Figure 1F**		
JM9513	*klp5-mNeonGreen:kanR klp6-mNeonGreen:hygR natR:Z:adh15-mCherry-atb2 cut11-mCherry:ura4* $h^?$	this study
JM9877	*mcp1::natR klp5-mNeonGreen:kanR klp6-mNeonGreen:hygR natR:Z:adh15-mCherry-atb2* $h^?$	this study
**Figure EV1A**		
JM7175	*klp5-GFP:kanR natR:Z:adh15-mCherry-atb2* h^-	this study
JM7129	*mcp1-GFP:hygR natR:Z:adh15-mCherry-atb2* h^-	this study
**Figure EV1B**		
JM2943	*klp5::kanR* h^+	T. Toda
JM3089	*klp6::ura4* h^-	T. Toda
JM7129	*mcp1-GFP:hygR natR:Z:adh15-mCherry-atb2* h^-	this study
JM7185	*mcp1-GFP:hygR natR:Z:adh15-mCherry-atb2 klp5::kanR h*	this study
JM7187	*mcp1-GFP:hygR natR:Z:adh15-mCherry-atb2 klp6::ura4 h*	this study
JM7208	*mcp1-GFP:hygR natR:Z:adh15-mCherry-atb2 klp5::kanR klp6::ura4 h*	this study
JM8520	*mcp1-GFP:hygR natR:Z:adh15-mCherry-atb2 klp5::kanR klp6::ura4 lys1::klp5:kanR his3::klp6:blaR* h^-	this study
JM8521	*mcp1-GFP:hygR natR:Z:adh15-mCherry-atb2 klp5::kanR klp6::ura4 lys1::kanR his3::blaR* h^-	this study
JM8583	*mcp1-GFP:hygR natR:Z:adh15-mCherry-atb2 klp5::kanR klp6::ura4 lys1::klp5(G277A,E299A):kanR his3::klp6(G296A,E298A):blaR* h^-	this study
**Figure EV1C**		
JM9325	*mcp1::natR lys1::nmt1-GFP-mcp1:ura4 klp5-13Myc:kanR* h^-	this study
**Figure EV1D**		
JM196	*cdc25-22* h^+	lab stock
JM8009	*mcp1::kanR cdc25-22* h^-	this study
JM5963	*klp5::natR klp6::ura4 cdc25-22* $h^?$	lab stock
JM9321	*mcp1::hygR cdc25-22 klp5::natR klp6::ura4* h^-	this study
**Figure EV1E**		
JM7175	*klp5-GFP:kanR natR:Z:adh15-mCherry-atb2* h^-	this study
JM7202	*mcp1::hygR klp5-GFP:kanR natR:Z:adh15-mCherry-atb2* h^-	this study
JM7177	*klp6-GFP:hygR natR:Z:adh15-mCherry-atb2* h^-	this study
JM7205	*mcp1::kanR klp6-GFP:hygR natR:Z:adh15-mCherry-atb2* h^-	this study
**Figure EV2A**		

JM3707	*fta3-GFP:kanR* h^-	X. He
JM3725	*sid4-TdTomato:hygR* h^+	I. Hagan
JM5061	*fta3-GFP:kanR sid4-tdTomato:hygR* h^-	lab stock
JM6143	*klp5::ura4 fta3-GFP:kanR sid4-tdTomato:hygR* $h^?$	lab stock
JM7141	*mcp1::natR fta3-GFP:kanR sid4-tdTomato:hygR* h^-	this study

Figure EV2B

JM2589	*cdc13-117:cdc13-GFP:LEU2* h^-	M. Yanagida
JM5785	*cdc13-117:cdc13-GFP:LEU2 sid4-TdTomato:hygR* h^-	this study
JM9138	*klp5::ura4 cdc13-117:cdc13-GFP:LEU2 sid4-TdTomato:hygR* h^+	this study
JM9131	*mcp1::natR cdc13-117:cdc13-GFP:LEU2 sid4-TdTomato:hygR* h^-	this study

Figure EV2C

JM2794	*ade6-M210 + Ch16(ade6-M216)* h^-	J-P. Javerzat
JM6343	*klp6::ura4 ade6-M210 + Ch16(ade6-M216)* $h^?$	this study
JM7181	*mcp1::natR ade6-M210 + Ch16(ade6-M216)* h^-	this study

Figure EV2D

JM2881	*dam1::kanR* h^+	lab stock
PR100	h^+	P. Russell
JM2943	*klp5::kanR* h^+	lab stock
JM7111	*mcp1::kanR* h^-	this study

Figure EV2E

JM3253	*klp5::kanR* h^-	lab stock
JM2958	*dam1:hygR* h^+	lab stock
JM2115	*dis2::ura4* h^+	M. Yanagida
JM8832	*bub3::hygR* h^+	lab stock
JM6523	*mcp1::natR* h^-	this study

Figure EV2F

JM9441	*klp5-mNeonGreen:kanR klp6-mNeonGreen:hygR natR:Z:adh15-mCherry-atb2* h^+	this study
JM9877	*mcp1::natR klp5-mNeonGreen:kanR klp6-mNeonGreen:hygR natR:Z:adh15-mCherry-atb2* $h^?$	this study

Figure EV2G

JM7129	*mcp1-GFP:hygR natR:Z:adh15-mCherry-atb2* h^-	this study

Figure 2A

JM9513	*klp5-mNeonGreen:kanR klp6-mNeonGreen:hygR natR:Z:adh15-mCherry-atb2 cut11-mCherry:ura4* $h^?$	this study
JM3010	*tea2-GFP:kanR* h^-	P. Nurse
JM8007	*tea2-GFP:kanR natR:Z:adh15-mCherry-atb2* h^-	this study

Figure 2B-C

JM8007	*tea2-GFP:kanR natR:Z:adh15-mCherry-atb2* h^-	this study
JM8070	*klp6::hygR tea2-GFP:kanR natR:Z:adh15-mCherry-atb2* $h^?$	this study
JM8053	*mcp1::hygR tea2-GFP:kanR natR:Z:adh15-mCherry-atb2* $h^?$	this study

Figure 2D

JM9441	*klp5-mNeonGreen:kanR klp6-mNeonGreen:hygR natR:Z:adh15-mCherry-atb2* h^+	this study
JM2895	*tea2::his3 his3-D1* h^-	P. Nurse
JM9961	*tea2::his3 klp5-mNeonGreen:kanR klp6-mNeonGreen:hygR natR:Z:adh15-mCherry-atb2 his3-D1* $h^?$	this study

Figure 2E-F

JM8018	*leu1-32::SV40-GFP-atb2:leu1* h^-	F. Chang
JM8427	*klp5::natR klp6::hygR leu1-32::SV40-GFP-atb2:leu1* h^-	this study
JM8334	*mcp1::kanR leu1-32::SV40-GFP-atb2:leu1* h^+	this study
JM8952	*tea2::his3 leu1-32::SV40-GFP-atb2:leu1 his3-D1* h^+	this study
JM9439	*tea2::his3 klp5::natR klp6::hygR leu1-32::SV40-GFP-atb2:leu1 his3-D1* $h^?$	this study
JM9202	*tea2::his3 mcp1::hygR leu1-32::SV40-GFP-atb2:leu1 his3-D1* h^+	this study
**Figure EV3A**		
JM9993	*tea2-GFP:kanR natR:Z:adh15-mCherry-atb2 cut11-mCherry:ura4* $h^?$	this study
JM8070	*klp6::hygR tea2-GFP:kanR natR:Z:adh15-mCherry-atb2* $h^?$	this study
JM8053	*mcp1::hygR tea2-GFP:kanR natR:Z:adh15-mCherry-atb2* $h^?$	this study
**Figure EV3B**		
JM8018	*leu1-32::SV40-GFP-atb2:leu1* h^-	F. Chang
JM8334	*mcp1::kanR leu1-32::SV40-GFP-atb2:leu1* h^+	this study
JM2893	*tip1::kanR* h^-	P. Nurse
JM8392	*tip1::kanR leu1-32::SV40-GFP-atb2:leu1* h^-	this study
JM8462	*mcp1::hygR tip1::kanR leu1-32::SV40-GFP-atb2:leu1* $h^?$	this study
JM2894	*tea1::ura4* h^-	P. Nurse
JM8929	*tea1::ura4 leu1-32::SV40-GFP-atb2:leu1* h^+	this study
JM8944	*mcp1::hygR tea1::ura4 leu1-32::SV40-GFP-atb2:leu1* h^+	this study
**Figure EV3C-D**		
JM9513	*klp5-mNeonGreen:kanR klp6-mNeonGreen:hygR natR:Z:adh15-mCherry-atb2 cut11-mCherry:ura4* $h^?$	this study
JM9961	*tea2::his3 klp5-mNeonGreen:kanR klp6-mNeonGreen:hygR natR:Z:adh15-mCherry-atb2 his3-D1* $h^?$	this study
**Figure EV3E**		
JM9513	*klp5-mNeonGreen:kanR klp6-mNeonGreen:hygR natR:Z:adh15-mCherry-atb2 cut11-mCherry:ura4* $h^?$	this study
JM10020	*mcp1::natR tea2::his3 klp5-mNeonGreen:kanR klp6-mNeonGreen:hygR natR:Z:adh15-mCherry-atb2 his3-D1* $h^?$	this study
**Figure 3A-B**		
JM9991	*tea2-GFP:kanR tip1-TdTomato:hygR* $h^?$	this study
**Figure 4A-B**		
JM9935	*klp5-mNeonGreen:kanR klp6-mNeonGreen:hygR tip1-TdTomato:hygR* $h^?$	this study
**Figure 4C**		
JM10018	*mcp1::natR klp5-mNeonGreen:kanR klp6-mNeonGreen:hygR tip1-TdTomato:hygR* $h^?$	this study
**Figure EV4**		
JM9935	*klp5-mNeonGreen:kanR klp6-mNeonGreen:hygR tip1-TdTomato:hygR* $h^?$	this study
**Figure EV5**		
JM10018	*mcp1::natR klp5-mNeonGreen:kanR klp6-mNeonGreen:hygR tip1-TdTomato:hygR* $h^?$	this study

All strains are *ura4-D18 leu1-32* unless otherwise stated.

Appendix Table S2. List of oligonucleotides used in this study

Klp5.tagW	CCTCATCTGGACACTATTGATCTGGAT
Klp5.tagX	GGGGATCCGTCGACCTGCAGCGTACGAGGTGGCTTTCTC TTCTTCGTT
Klp5.tagY	GTTTAAACGAGCTCGAATTCATCGATCGGATAAGCTTGAT GATATGA
Klp5.tagZ	GGTGGCAGACATATATGTACGCTT
Klp6.tagW	GTGGAAAAATCTTTAGATAAACATAAT
Klp6.tagX	GGGGATCCGTCGACCTGCAGCGTACGAAGCATTAGGAGT ATTCTCAGTCCCGCC
Klp6.tagY	GTTTAAACGAGCTCGAATTCATCGATGCTACTTATGCTACT TATGATTTAGCATTTCAT
Klp6.tagZ	GGTCAATCATTAACCCGATTAAAT
Mcp1.delW	AGCATTTTTTAACTATAATAAGC
Mcp1.delX	GGGGATCCGTCGACCTGCAGCGTACGAAATATCTTATGAT TTTAACATTAAAAACAG
Mcp1.delY	GTTTAAACGAGCTCGAATTCATCGATTTTGTCTCTCGTTAC TCTACAATATTT
Mcp1.delZ	GGCTTAACGAATTCCTTGGATACCA
Mcp1.tagW	CCCAAAGGTGAATTTACTAATTCACTT
Mcp1.tagX	GGGGATCCGTCGACCTGCAGCGTACGATAGAAAAGTAGG TAGTTTAGAACCGGGGATCCGTCGACCTGCAGCGTACGA
Mcp1.tagY	GTTTAAACGAGCTCGAATTCATCGATATTAATGTATGAATA TAATGAAATAATTCGTAA
Mcp1.tagZ	CAATTTTCTTTGGTGTCCTGACAAAGCGT
Mcp1.pLys1U.FW	AGTAGTCGACGTCCTCTTTTTTTTAACGGGA
Mcp1.pLys1U.RV	TCATGGATCCCAAAGCACTTAAAATTAAAAAA
