# Supplementary material for: Opposing kinesin complexes queue at plus tips to ensure microtubule catastrophe at cell ends
Source: EMBO Rep. 2018 Sep 11;19(11):e46196. doi: 10.15252/embr.201846196 (PMC6216294; doi:10.15252/embr.201846196)
Supplement: Supplementary file 2 — Expanded View Figures PDF [file EMBR-19-e46196-s002.pdf]

Expanded View Figures

Figure EV1. Mcp1 interacts with Klp5/Klp6 and requires its motor activity to accumulate at microtubule plus ends but is dispensable for cellular polarity control.

A Upper panels showing fluorescently tagged microtubules (MT) and either Klp5-GFP (left panel) or Mcp1-GFP (right panel) imaged every 4.8 s. Dashed yellow line indicates the cell end. Scale bar, 5 μm. Lower panels show the mean intensity ± s.d. of either Klp5-GFP (left panel, $n = 20$) or Mcp1-GFP (right panel, $n = 20$) at the plus ends of iMTs.

B Plots show the mean distance moved over time of GFP puncta associated with growing iMTs from each of the indicated backgrounds. Error bars show standard deviation from five replicates.

C Log phase cultures of *GFP-mcp1 klp5-13Myc* cells were harvested and lysed. Proteins were immunoprecipitated from 2 mg of whole cell extract (WCE) using rabbit α-GFP antibodies (I) or pre-immune control (PI), migrated by SDS–PAGE and probed with either sheep α-GFP or mouse α-Myc antibodies. 50 μg of WCE was run and immunoblotted for comparison.

D Images show *cdc25-22* cells (left panel) or *cdc25-22 Δklp5 Δklp6* cells (right panel) arrested at the restrictive temperature (35.5°C) for 6 h. Scale bar, 5 μm. Cellular curvature was quantitated, as in the schematic, by measuring both the cell length (length, L) and the distance between cell ends (Euclidean distance, E) and then calculating the ratio (L:E). These ratios, converted to percentages, are displayed on the plot, with red lines showing the mean value. ~850 cells were measured for each strain.

E Log phase cultures of cells expressing *klp5-GFP* (left panels) or *klp6-GFP* (right panel) in control or *Δmcp1* cells were lysed and proteins extracted. 50 μg of each was then migrated by SDS–PAGE, transferred to nitrocellulose membrane and probed with both α-GFP to determine protein level and α-Tat1 to use tubulin as a loading control.

Source data are available online for this figure.

© 2018 The Authors

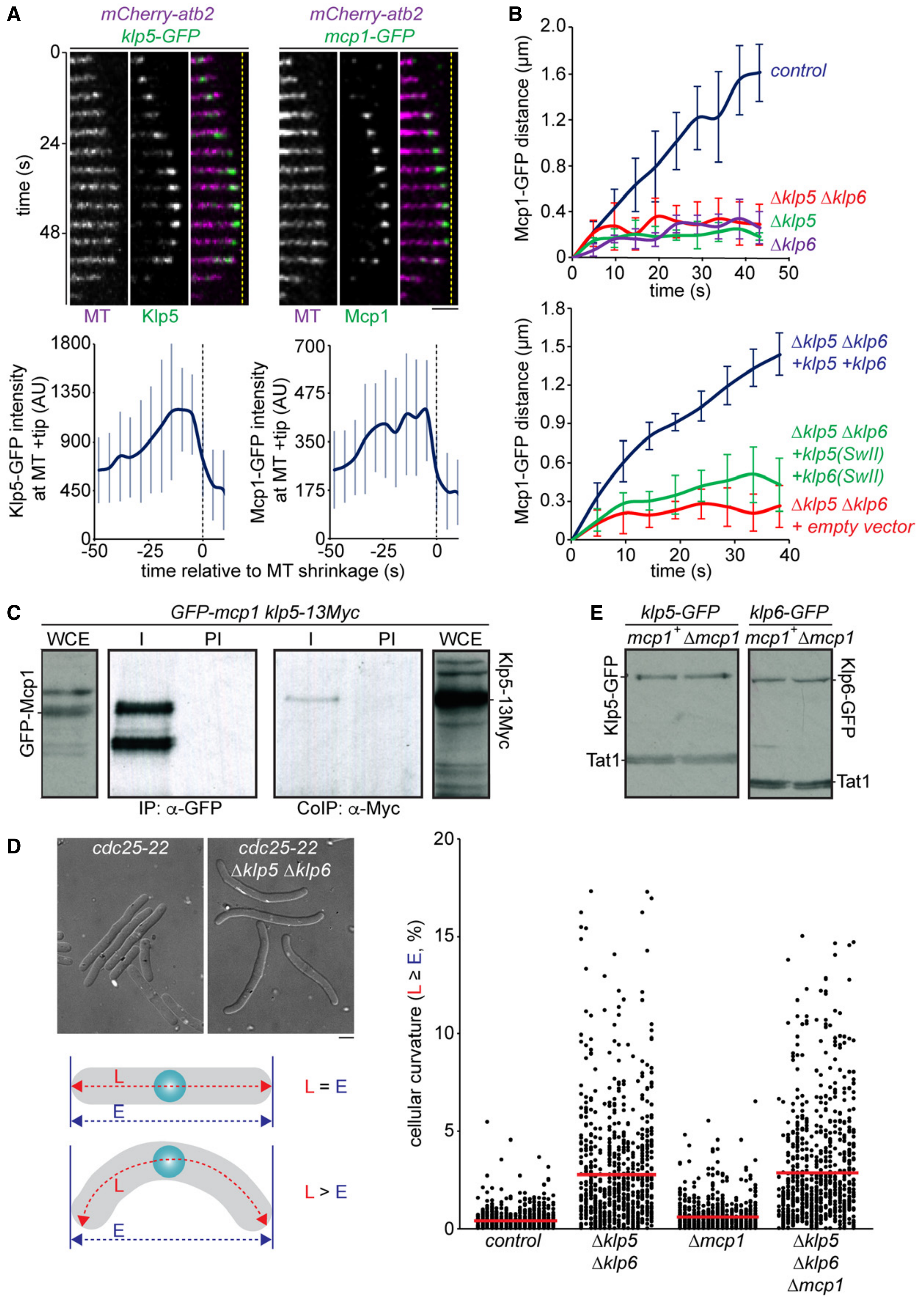

Figure EV1.

© 2018 The Authors

Figure EV2. Mcp1 does not control the mitotic functions of Klp5/Klp6.

- A Log phase cultures of control, *Δklp5* or *Δmcp1* cells expressing fluorescently tagged kinetochore (Fta3) and spindle pole body (Sid4) proteins were imaged. The proportion of pre-anaphase mitotic cells with unseparated kinetochore pairs between poles was determined (PM & M).
- B Log phase cultures of control, *Δklp5* or *Δmcp1* cells expressing fluorescently tagged cyclin B (Cdc13) and Sid4 were imaged. The proportion of cells with Cdc13-GFP on separated poles and spindles was determined.
- C Log phase cultures of control, *Δklp5* or *Δmcp1* cells expressing *ade6-M210* and carrying the *Ch16(ade6-M216)* mini-chromosome were grown in media lacking adenine and then individual cells plated onto media with minimal adenine. After 3 days, the proportion of cells that had formed colonies that were ≥ 50% red sectored, having lost the mini-chromosome in the first mitotic division, was determined.
- D Log phase cultures of control, *Δklp5*, *Δdam1* or *Δmcp1* cells were serially diluted onto plates containing the indicated concentrations of the MT poison thiabendazole (TBZ) and grown for 3 days. Cells lacking Klp5 have elevated resistance to TBZ, whereas cells lacking Dam1, a component of the DASH complex, display enhanced sensitivity to TBZ.
- E Viability of strains lacking Dam1, Dis2 or Bub3 without either Klp5 or Mcp1.
- F Mitotic cells expressing fluorescently tagged MTs and Klp5/Klp6 in the presence (left panels) or absence (right panels) of Mcp1. Scale bar, 2 μm.
- G Mitotic cell expressing fluorescently tagged MTs and Mcp1. Scale bar, 2 μm.

Data information: In (A–C), the mean from three independent experiments is plotted + s.d.
Source data are available online for this figure.

© 2018 The Authors

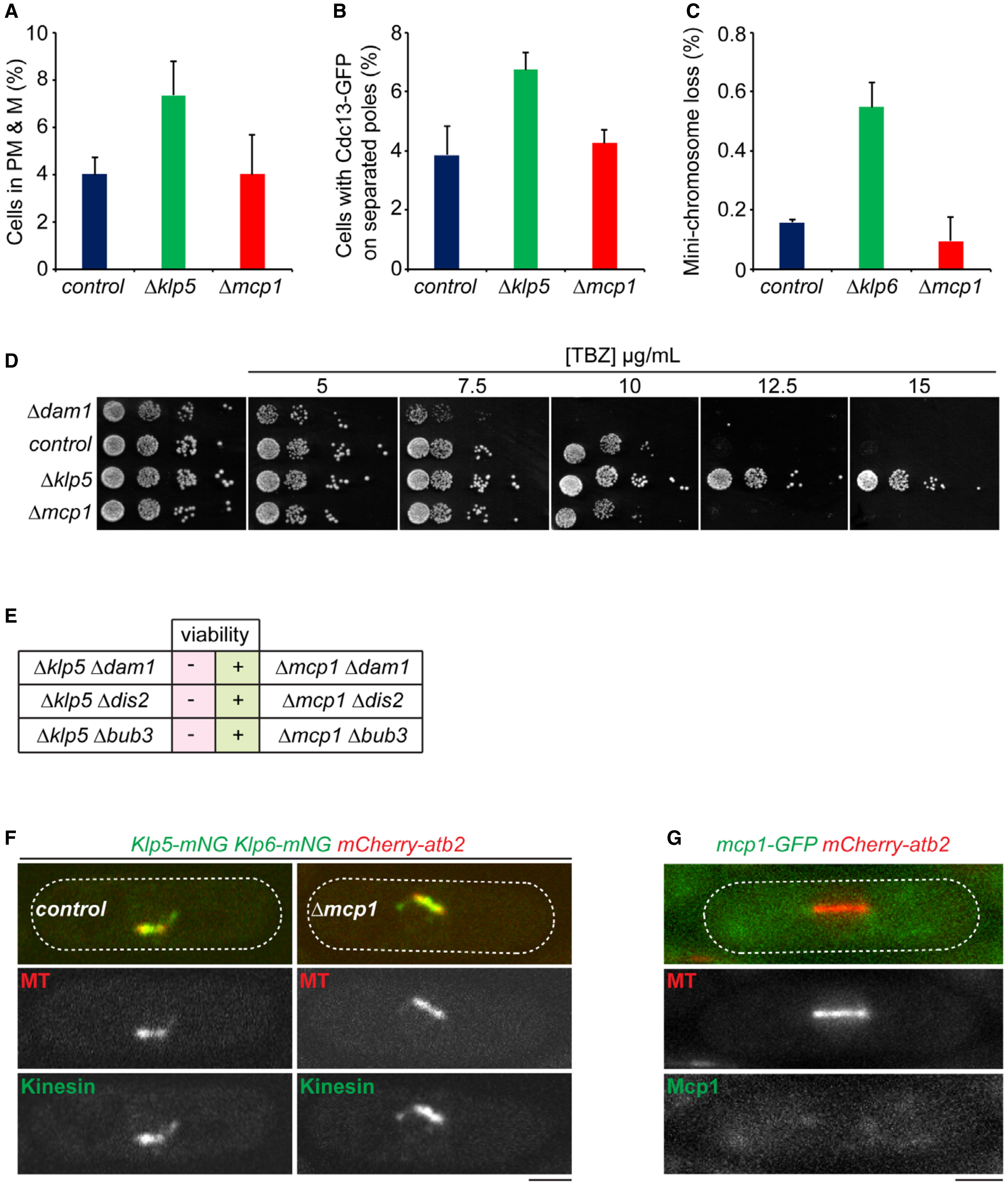

Figure EV2.

 © 2018 The Authors

Figure EV3. Effect of mutants on kinesin accumulation at plus ends and microtubule dwell time.

A Box plots show data from mixing experiments comparing the intensity of fluorescently tagged Tea2 on MT plus ends between either Δ*klp6* (*n* = 50) and control cells (*n* = 49) (left panel) or between Δ*mcp1* (*n* = 50) and control cells (*n* = 50; right panel).

B Cells expressing fluorescently tagged tubulin were imaged every 5 s and the dwell time of ~100 individual iMTs for each condition recorded within the final 1.1 μm of the cell. Red bars signify the mean.

C Intensity of Klp5/Klp6 at MT plus ends relative to the time of MT shrinkage was quantified from multiple kymographs (*n* = 16) of *Δtea2* cells (top panel). Box plots (bottom panel) from mixing experiments comparing the amount of fluorescently tagged Klp5/Klp6 localised either in the nucleus or on MT plus ends before shrinkage between control (*n* = 37) and Δ*tea2* (*n* = 37) cells.

D Log phase cells expressing fluorescently tagged MTs were imaged, and the levels of fluorescently tagged Klp5/Klp6 at the plus ends of growing MTs were determined. Measurements (*control*, *n* = 100; Δ*tea2*, *n* = 100) were plotted against microtubule (MT) length and second-order polynomial curves fitted to the data. Dashed line indicates the estimated division between MT growth, where MT lifetime directly correlates to MT length, and MT dwell, where MT lifetime no longer directly correlates to MT length due to MT pausing at cell ends.

E Experiments as for (C) but with kymographs of *Δmcp1 Δtea2* cells (*n* = 24) (top panel) and for box plots (bottom panel) between control (*n* = 50) and Δ*mcp1* Δ*tea2* (*n* = 50) cells.

Data information: In (C and E, upper panels), data are presented as mean ± s.d. *$P < 0.001$, **$P < 0.01$, n.s. (non-significant) $P > 0.05$ (Kolmogorov–Smirnov test). In (A, C and E), boxes show the interquartile range with the median represented between the lower and upper quartiles, and whiskers show the highest and lowest values.
Source data are available online for this figure.

© 2018 The Authors

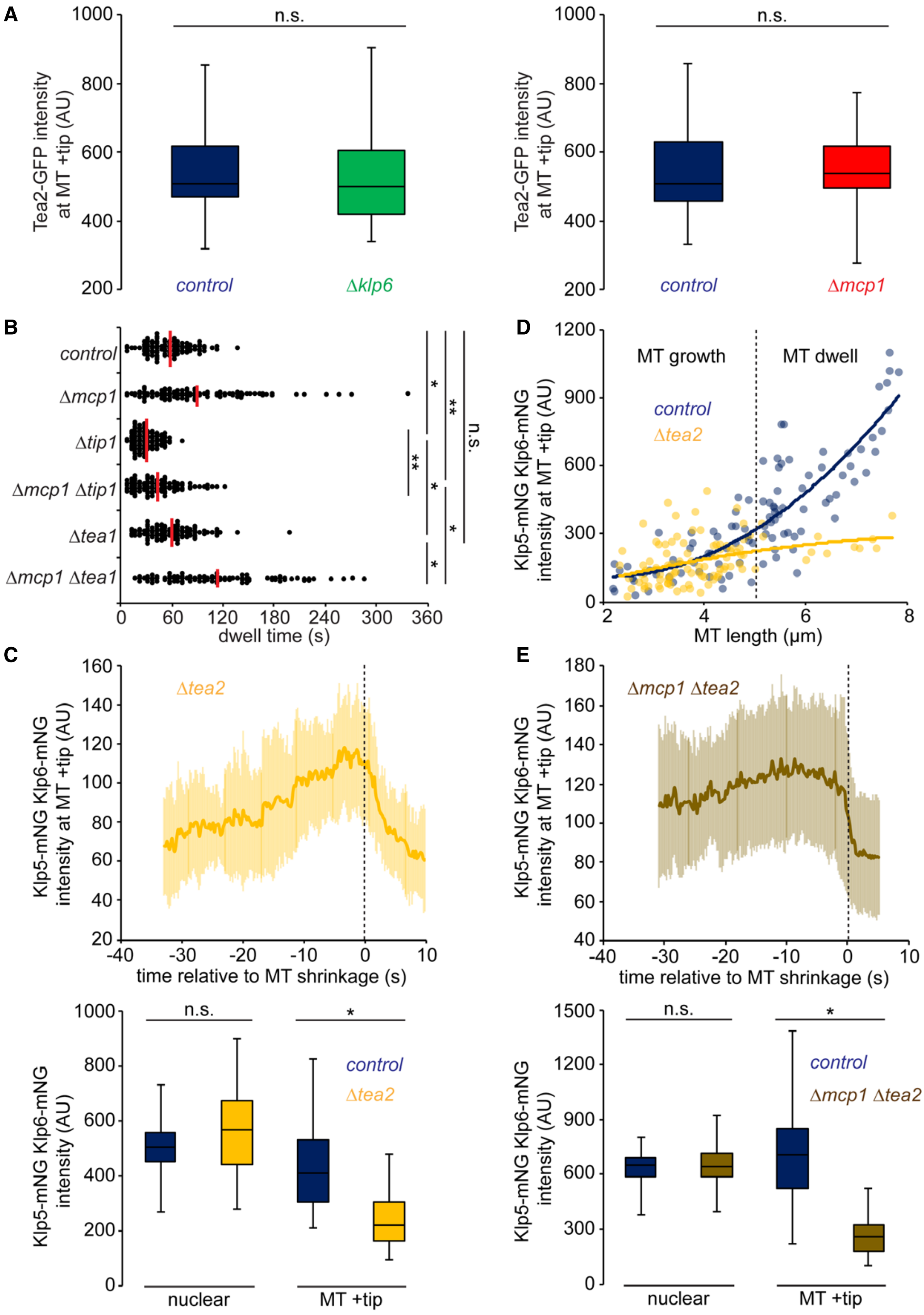

Figure EV3.

© 2018 The Authors

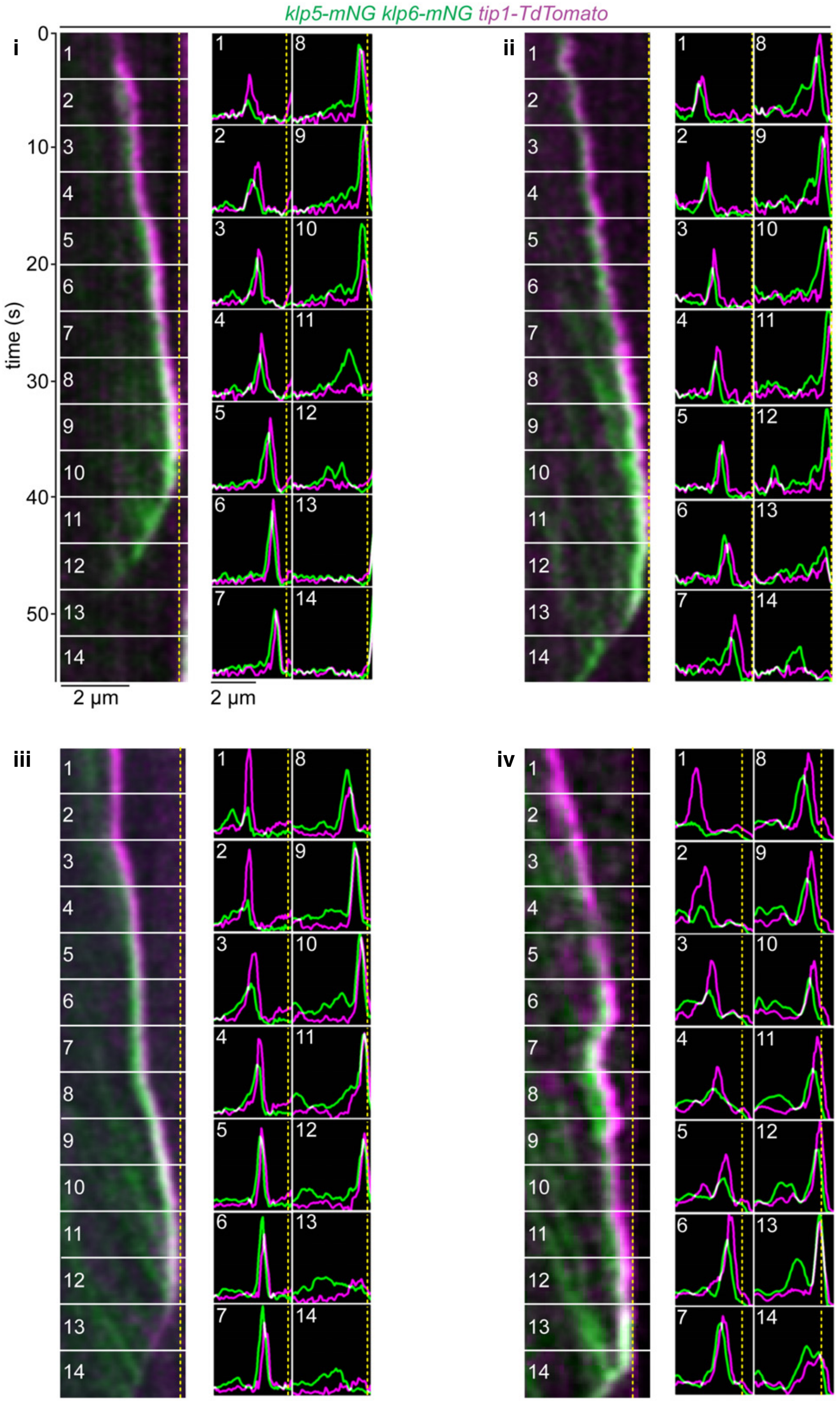

Figure EV4. Location of Klp5/Klp6 and Tea2/Tip1 at the plus end during microtubule growth, dwell and shrinkage.
Four additional kymographs (*i–iv*) from cells expressing fluorescently tagged Klp5/Klp6 (green) co-imaged with fluorescently tagged Tip1 (magenta). Dashed yellow lines indicate cell ends. Associated plots show both the relative fluorescence intensity and position for Klp5/Klp6 (green) and Tip1 (magenta) corresponding to the numbered sections of the kymographs.

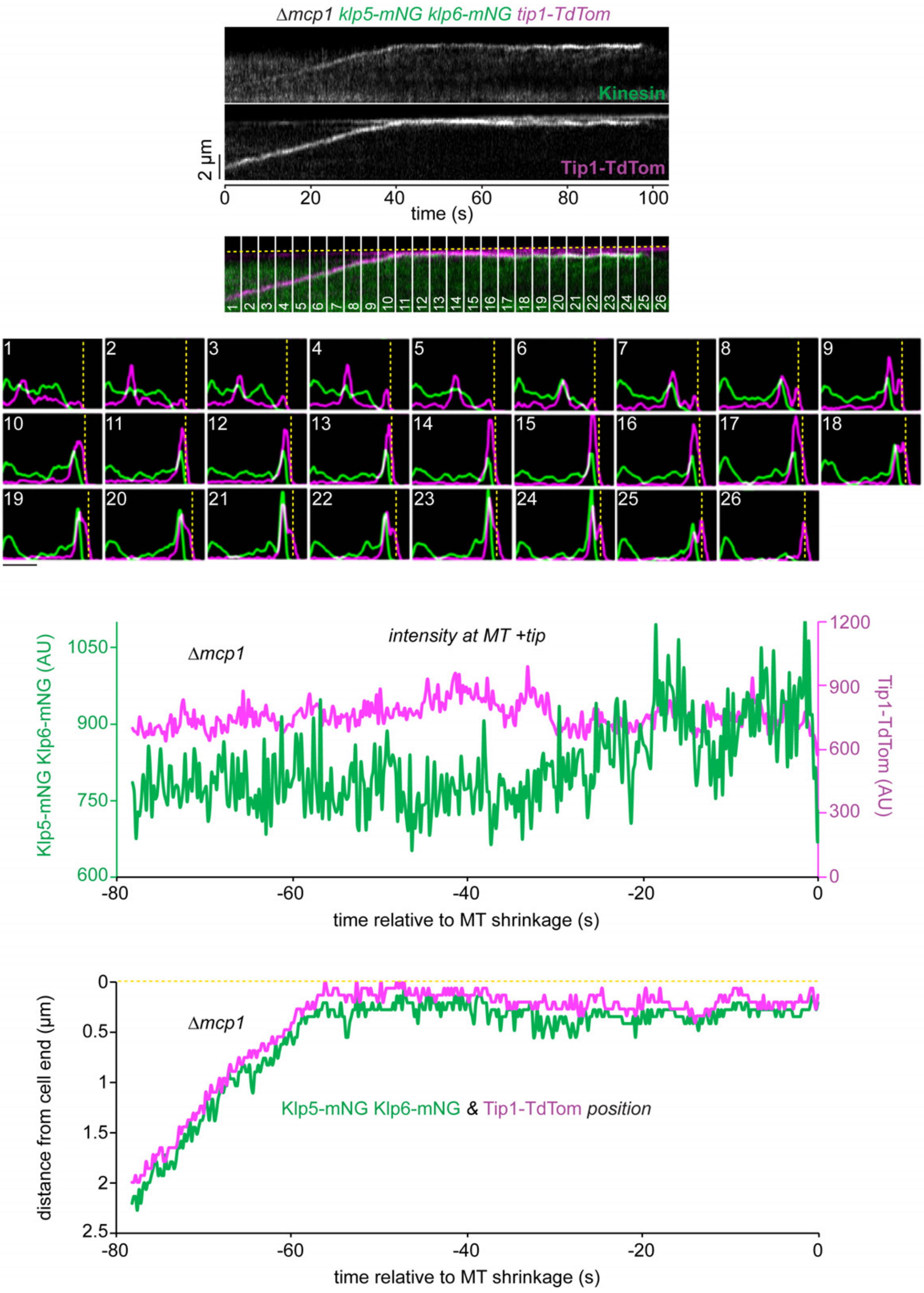

Figure EV5. Klp5/Klp6 queues behind Tea2 for longer in the absence of Mcp1.

Kymograph (top panels) showing fluorescently tagged Klp5/Klp6 (Kinesin) co-imaged with fluorescently tagged Tip1 (Tip1-Tdtom) in the absence of Mcp1. Dashed yellow line indicates the cell end. Plots (second top panel) showing both the relative fluorescence intensity and position for Klp5/Klp6 (green) and Tip1 (magenta) corresponding to the numbered sections of the kymograph. Scale bar, 2 µm. Data quantitated from this kymograph by extracting the maximal intensity pixel value at the MT plus end over time for both Klp5/Klp6 and Tip1 (second bottom panel) and by plotting the distance that these pixels are from the cell end relative to MT shrinkage (bottom panel).

© 2018 The Authors
