# Supplementary material for: Opposing kinesin complexes queue at plus tips to ensure microtubule catastrophe at cell ends
Source: EMBO Rep. 2018 Sep 11;19(11):e46196. doi: 10.15252/embr.201846196 (PMC6216294; doi:10.15252/embr.201846196)
Supplement: Supplementary file 4 — Source Data for Expanded View [file EMBR-19-e46196-s006.zip › embr201846196-sup-0008-SDataFigEV3ACE.pdf]

Figure EV3 - source data

A - Tea2 fluorescence intensity

	mean ± standard deviation (s)	number of observations	Kolmogorov-Smirnov test (p)
control	531.6 ± 111.4	49	0.1248
Δklp6	523.9 ± 132.9	50	
control	550.3 ± 132.8	50	0.1122
Δmcp1	545.7 ± 107.5	50	

B - iMT dwell time at cell ends

	mean ± standard deviation (s)	number of observations	Kolmogorov-Smirnov test (p)					
control	54.2 ± 25.2	104						
Δmcp1	87.5 ± 61.3	100	2.96×10^{-10}					
Δtip1	27.7 ± 13.7	100				0.005319		0.918
Δmcp1 Δtip1	41.05 ± 26.4	100		8.6×10^{-11}	0.003861			
Δtea1	57.3 ± 30.2	104					2.96×10^{-13}	
Δmcp1 Δtea1	111.2 ± 64.5	94	6.9×10^{-10}					

C - Klp5/Klp6 fluorescence intensity

		mean ± standard deviation (AU)	number of observations	Kolmogorov-Smirnov test (p)
nuclear	*control*	503.2 ± 104.4	37	0.134
	Δtea2	553.7 ± 152.4	37	
MT +tip	*control*	415.6 ± 137.7	37	1.33×10^{-5}
	Δtea2	238.6 ± 99.3	37	

E - Klp5/Klp6 fluorescence intensity

		mean ± standard deviation (AU)	number of observations	Kolmogorov-Smirnov test (p)
nuclear	*control*	535.9 ± 88.6	50	0.3927
	Δmcp1 Δtea2	548.78 ± 115.0	50	
MT +tip	*control*	613.5 ± 248.2	50	$<2.2 \times 10^{-16}$
	Δmcp1 Δtea2	160.1 ± 91.0	50	
