# Supplementary material for: Opposing kinesin complexes queue at plus tips to ensure microtubule catastrophe at cell ends
Source: EMBO Rep. 2018 Sep 11;19(11):e46196. doi: 10.15252/embr.201846196 (PMC6216294; doi:10.15252/embr.201846196)
Supplement: Supplementary file 4 — Source Data for Expanded View [file EMBR-19-e46196-s006.zip › embr201846196-sup-0006-SDataFigEV1D.pdf]

Figure EV1 - source data

D - Cellular curvature

	mean ± standard deviation (%)	number of observations
control	0.38 ± 0.48	>280 cells x3
Δklp5 Δklp6	2.72 ± 3.03	>280 cells x3
Δmcp1	0.53 ± 0.79	>280 cells x3
Δklp5 Δklp6 Δmcp1	2.81 ± 2.80	>280 cells x3
