# Supplementary material for: Opposing kinesin complexes queue at plus tips to ensure microtubule catastrophe at cell ends
Source: EMBO Rep. 2018 Sep 11;19(11):e46196. doi: 10.15252/embr.201846196 (PMC6216294; doi:10.15252/embr.201846196)
Supplement: Supplementary file 4 — Source Data for Expanded View [file EMBR-19-e46196-s006.zip › embr201846196-sup-0007-SDataFigEV2AC.pdf]

Figure EV2 - source data

A - Proportion of *dad1-GFP sid4-TdTomato* cells in prometaphase and metaphase (PM & M)

	mean ± standard deviation (%)	number of observations
control	4.0 ± 0.72	>500 cells x3
Δklp5	7.3 ± 1.44	>500 cells x3
Δmcp1	4.0 ± 1.68	>500 cells x3

B - Proportion of cells with Cdc13-GFP on separated poles

	mean ± standard deviation (%)	number of observations
control	3.6 ± 1.27	>500 cells x3
Δklp5	6.9 ± 0.71	>500 cells x3
Δmcp1	4.1 ± 0.57	>500 cells x3

C - Proportion of cells that lose mini-chromosome in first mitotic division

	mean ± standard deviation (%)	number of observations
control	0.16 ± 0.02	>1000 cells x3
Δklp6	0.58 ± 0.08	>1000 cells x3
Δmcp1	0.07 ± 0.10	>1000 cells x3
