# Supplementary material for: Opposing kinesin complexes queue at plus tips to ensure microtubule catastrophe at cell ends
Source: EMBO Rep. 2018 Sep 11;19(11):e46196. doi: 10.15252/embr.201846196 (PMC6216294; doi:10.15252/embr.201846196)
Supplement: Supplementary file 6 — Source Data for Figure 1 [file EMBR-19-e46196-s004.pdf]

Figure 1 - source data

B - iMT dwell time at cell ends

	mean ± standard deviation (s)	number of observations	Kolmogorov-Smirnov test (p)
control	54.2 ± 25.2	104	5.154×10^{-5}
Δklp5 Δklp6	84.6 ± 58.9	97	0.8146
Δmcp1	87.5 ± 61.3	100	0.791
Δklp5 Δklp6 Δmcp1	96.4 ± 65.4	102	

D - MT growth speed & Klp5/Klp6 speed on MT lattice

		mean ± standard deviation (nm/s)	number of observations	Kolmogorov-Smirnov test (p)
MT growth speed	*control*	69.0 ± 19.9	16	0.7648
	Δmcp1	68.8 ± 22.8	11	5.96×10^{-9}
Klp5/Klp6 speed on MT lattice	*control*	133.5 ± 27.6	44	1.258×10^{-4}
	Δmcp1	168.0 ± 36.7	32	

F - Klp5/Klp6 fluorescence intensity

		mean ± standard deviation (AU)	number of observations	Kolmogorov-Smirnov test (p)
nuclear	*control*	787.5 ± 222.0	44	0.5435
	Δmcp1	816.7 ± 212.8	45	
MT +tip	*control*	495.9 ± 214.7	64	1.287×10^{-7}
	Δmcp1	234.4 ± 177.2	35	
