# Supplementary material for: Opposing kinesin complexes queue at plus tips to ensure microtubule catastrophe at cell ends
Source: EMBO Rep. 2018 Sep 11;19(11):e46196. doi: 10.15252/embr.201846196 (PMC6216294; doi:10.15252/embr.201846196)
Supplement: Supplementary file 7 — Source Data for Figure 2 [file EMBR-19-e46196-s005.pdf]

Figure 2 - source data

E - iMT dwell time at cell ends

	mean ± standard deviation (s)	number of observations	Kolmogorov-Smirnov test (p)			
control	54.2 ± 25.2	104				
Δklp5 Δklp6	84.6 ± 58.9	97	4.159×10^{-11}			
Δmcp1	87.5 ± 61.3	100				0.01274
Δtea2	28.7 ± 15.6	100				
Δklp5 Δklp6 Δtea2	43.0 ± 24.3	93		1.908×10^{-5}		
Δmcp1 Δtea2	42.15 ± 23.2	100			0.2242	

F - Distance from cell end when MT undergoes shrinkage

	mean ± standard deviation(µm)	number of observations
control	0.21 ± 0.085	20
Δklp5 Δklp6	0.34 ± 0.43	20
Δmcp1	0.17 ± 0.11	20
Δtea2	1.71 ± 1.29	20
Δklp5 Δklp6 Δtea2	0.50 ± 0.71	20
Δmcp1 Δtea2	0.45 ± 0.45	20
